# 5-Keto-D-Fructose, a Natural Diketone and Potential Sugar Substitute, Significantly Reduces the Viability of Prokaryotic and Eukaryotic Cells

**DOI:** 10.3389/fmicb.2022.935062

**Published:** 2022-06-21

**Authors:** Marcel Hövels, Nicole Gallala, Samara Lisa Keriakes, Anna Paulina König, Jacqueline Schiessl, Tobias Laporte, Konrad Kosciow, Uwe Deppenmeier

**Affiliations:** ^1^Institute for Microbiology and Biotechnology, University of Bonn, Bonn, Germany; ^2^German Aerospace Center (DLR), Institute for the Protection of Terrestrial Infrastructures, Sankt Augustin, Germany

**Keywords:** 5-keto-D-fructose, toxicology, Maillard reaction, sugar substitutes, HT-29 cell line

## Abstract

5-Keto-D-fructose (5-KF) is a natural diketone occurring in micromolar concentrations in honey, white wine, and vinegar. The oxidation of D-fructose to 5-KF is catalyzed by the membrane-bound fructose dehydrogenase complex found in several acetic acid bacteria. Since 5-KF has a sweetening power comparable to fructose and is presumably calorie-free, there is great interest in making the diketone commercially available as a new sugar substitute. Based on a genetically modified variant of the acetic acid bacterium *Gluconobacter oxydans* 621H, an efficient process for the microbial production of 5-KF was recently developed. However, data on the toxicology of the compound are completely lacking to date. Therefore, this study aimed to investigate the effect of 5-KF on the viability of prokaryotic and eukaryotic cells. It was found that the compound significantly inhibited the growth of the gram-positive and gram-negative model organisms *Bacillus subtilis* and *Escherichia coli* in a concentration-dependent manner. Furthermore, cell viability assays confirmed severe cytotoxicity of 5-KF toward the colon cancer cell line HT-29. Since these effects already occurred at concentrations of 5 mM, the use of 5-KF in the food sector should be avoided. The studies performed revealed that in the presence of amines, 5-KF promoted a strong Maillard reaction. The inherent reactivity of 5-KF as well as the Maillard products formed could be the trigger for the observed inhibition of prokaryotic and eukaryotic cells.

## Introduction

A growing body of epidemiological evidence supports the claim that excessive sugar consumption elevates the abundance of first-world diseases, such as insulin resistance, type 2 diabetes mellitus, obesity, metabolic syndrome, hypertension, and cardiovascular diseases (Johnson et al., 2007; Dekker et al., 2010; Hu and Malik, 2010; Malik et al., 2010; Tappy and Lê, 2010; Aragno and Mastrocola, 2017). This causality and the growing awareness among consumers about the adverse effects of excessive sugar consumption are leading to an increasing demand for suitable sugar substitutes. Over the past decades, numerous sugar alcohols and synthetic sweeteners have been approved as sugar substitutes. Due to their reduced or non-existent caloric value, these substances are counteracting the spread of sugar-associated first-world diseases (Kroger et al., 2006; Chattopadhyay et al., 2014). However, many of the available sugar substitutes are characterized by unpleasant off-tastes (Schiffman et al., 1995) and have been shown to induce laxative effects, flatulence (Grembecka, 2015), or undesirable modulation of the gut microbiota (Suez et al., 2014). Unpleasant off-tastes are more prevalent among synthetic sweeteners such as saccharin or cyclamate, which taste bitter or metallic, for example (Chattopadhyay et al., 2014). Laxative effects and flatulence, on the other hand, are more likely caused by sugar alcohols due to their higher intake rates and water-pulling properties (Mäkinen, 2016).

The search for substances that provide a pleasant and sufficient sweetening power without triggering the mentioned side effects led to the discovery of 5-keto-D-fructose (5-KF, 5-ketofructose, 5-oxofructose, 5-dehydro-D-fructose, threo-2,5-hexodiulose), a natural diketone found in honey, white wine, and vinegar in low concentrations (Burroughs and Sparks, 1973; Schiessl et al., 2021). This potential sugar substitute can be produced by enzymatic oxidation of D-fructose, a reaction catalyzed by the membrane-bound fructose dehydrogenase complex (FDH_*SCL*_) found in a variety of acetic acid bacteria (Mowshowitz et al., 1974; Ameyama et al., 1981; Kawai et al., 2013). After Terada et al. (1960) already reported a sweet taste of 5-KF in the 1960s, the compound was recently evaluated in a dedicated sensory study (Herweg et al., 2018). A trained tasting panel could not distinguish between iso-sweet solutions of D-fructose (60 mM) and 5-KF (70 mM), indicating that 5-KF is almost as sweet as D-fructose. In addition, the sensory evaluation revealed overall high taste quality and total absence of off-tastes for 5-KF (Herweg et al., 2018). Due to these promising features, great efforts have been made to develop a biotechnological production process for 5-KF based on suitable acetic acid bacteria. In this context, heterologous production of the FDH_*SCL*_ encoded in the genome of the strictly aerobic alphaproteobacterium *Gluconobacter* (*G.*) *japonicus* NBRC3260 in the closely related strain *G. oxydans* 621H proved to be most efficient. Fed-batch fermentation of the FDH_*SCL*_ overproducing strain *G. oxydans fdh* resulted in a 5-KF concentration of 489 g L^–1^ at a conversion efficiency of 98% (Herweg et al., 2018; Siemen et al., 2018). These results could pave the way for large-scale production and commercial distribution of the 5-KF (Herweg et al., 2020). However, dedicated analyses of the compound’s uptake, distribution, metabolism, excretion, and corresponding toxicological studies are required prior to the application of 5-KF as a novel food compound. Although data are relatively sparse in this regard, 5-KF has been shown to be a suitable substrate for both rat and bovine liver fructokinase, leading to the formation of 5-keto-D-fructose-1-phosphate (Englard et al., 1972). This monophosphate was found to be a potent competitive inhibitor of the D-fructose-1-phosphate cleavage catalyzed by liver aldolase (Englard et al., 1972). Concerning microbial degradation, it was demonstrated that 5-KF could not be degraded by 15 members of the most common and abundant intestinal microorganisms (Schiessl et al., 2021). Thus, the sugar derivative seems to be an unsuitable growth substrate for prokaryotes in the human intestine. Besides, environmental bacteria such as *Tatumella morbirosei*, *Clostridium pasteurianum*, and *Gluconobacter* sp. were identified as capable of 5-KF consumption. Environmental accumulation, a phenomenon that has been reported recently for multiple synthetic sweeteners (Sang et al., 2014), is therefore unlikely in the case of 5-KF.

To further elucidate the effect of 5-KF on prokaryotic and eukaryotic cells, the compound was produced in larger quantities in this work, purified, and used for growth experiments and toxicological studies. The obtained results indicate that diketone significantly reduces the viability of gram-negative and gram-positive bacteria as well as HT-29 cells at concentrations ≥ 5 mM. The use of 5-KF in the food sector should therefore be reconsidered.

## Materials and Methods

### Chemicals

Chemicals and reagents were purchased from Sigma-Aldrich (St. Louis, US), Carl Roth GmbH & Co., KG (Karlsruhe, Germany), PAN-Biotech GmbH (Aidenbach, Germany), and Promega GmbH (Walldorf, Germany).

### Cultivation of Prokaryotic and Eukaryotic Cells

#### Shake Flask Cultivation of Microbial Strains

*G. oxydans* strains (Table 1) were cultured aerobically in shake flasks at 30^°^C, and 180 rpm in YMF-medium (6 g L^–1^ yeast extract, 0.6 g L^–1^ D-mannitol, and 0.06 g L^–1^ D-fructose) supplemented with 50 μg mL^–1^ cefoxitin. Cultures of the plasmid-bearing strain *G. oxydans fdh* were additionally supplemented with 50 μg mL^–1^ kanamycin.

**TABLE 1:** Overview of utilized strains and cell lines.

Strain/cell line	Genotype/description	Origin
*G. oxydans* 621H Δ*hsdR*	Δ*hsdR* (Δ*gox2567*) derivative of *G. oxydans* 621H (DSM 2343); Cef*^R^*	S. Bringer-Meyer, Forschungszentrum Jülich, Germany
*G. oxydans* 621H Δ*hsdR* pBBR1-p264-fdhSLC-ST*^a^* (referred to as *G. oxydans fdh*)	*G. oxydans* 621H Δ*hsdR* expressing the *fdh*_*SCL*_-operon (GenBank accession: AB728565.1) from *G. japonicus* NBRC3260 under control of the constitutive p264 promotor; Cef*^R^*, Kan*^R^*	Siemen et al., 2018
*E. coli* DSM 498	Type strain	DSMZ, Braunschweig, Germany,
*B. subtilis* DSM 10	Type strain	DSMZ, Braunschweig, Germany,
HT-29 ATCC HTB-38	Colon cell line with epithelial morphology	ATCC, Manassas, US

*^a^Naming of this strain is inconsistent within the literature. The existing synonyms G. oxydans 621H ΔhsdR pBBR1-p264-fdhSLC-ST (Siemen et al., 2018), G. oxydans 621H ΔhsdR pBBR1p264-FDH-Strep (Herweg et al., 2018), G. oxydans fdh (Hoffmann et al., 2020), and G. oxydans 621H pBBR1p264-fdhSCL-ST (Battling et al., 2020) are all describing the same strain.*

Precultures of *Escherichia* (*E.*) *coli* DSM 498 were cultivated aerobically in shake flasks at 37^°^C and 180 rpm in modified Wilms-MOPS-medium (Wilms et al., 2001). The mineral medium consisted of 6.98 g L^–1^ (NH_4_)_2_SO_4_, 3 g L^–1^ K_2_HPO_4_, 2 g L^–1^ Na_2_SO_4_, 41.85 g L^–1^ 3-(*N*-Morpholino)-propane sulfonic acid (MOPS), 0.5 g L^–1^ MgSO_4_ × 7 H_2_O, 0.01 g L^–1^ thiamine hydrochloride, 1 mL L^–1^ trace element solution (0.54 g L^–1^ ZnSO_4_ × 7 H_2_O, 0.48 g L^–1^ CuSO_4_ × 5 H_2_O, 0.3 g L^–1^ MnSO_4_ × H_2_O, 0.54 g L^–1^ CoCl_2_ × 6 H_2_O, 41.76 g L^–1^ FeCl_3_ × 6 H_2_O, 1.98 g L^–1^ CaCl_2_ × 2 H_2_O, 33.4 g L^–1^ Na_2_EDTA × 2 H_2_O). Glucose was added as the carbon source at a final concentration of 20 g L^–1^. The pH was adjusted to 7.5 using 1 M NaOH.

Precultures of *Bacillus* (*B.*) *subtilis* DSM 10 were cultivated aerobically in shake flasks at 30^°^C and 180 rpm in Spore minimal medium (Demain, 1958). To prepare the medium, 100 mL of a salt solution consisting of 30 g L^–1^ K_2_HPO_4_, 10 g L^–1^ KH_2_PO_4_, 5 g L^–1^ NH_4_Cl, 1 g L^–1^ NH_4_NO_3_, 1 g L^–1^ Na_2_SO_4_, 0.1 g L^–1^ MgSO_4_ × 7 H_2_O, 0.01 g L^–1^ MnSO_4_ × 4 H_2_O, 0.01 g L^–1^ FeSO_4_ × 7 H_2_O, and 0.005 g L^–1^ CaCl_2_ were added to 900 mL of a solution containing 11.1 g L^–1^ glucose, 1 g L^–1^ L-alanine, 1.63 g L^–1^ L-glutamic acid, and 1.47 g L^–1^ L-asparagine. The pH of both solutions was adjusted to 6.9 with 1 M NaOH and 1 M HCl before autoclaving separately.

#### Plate-Reader Cultivation of Microbial Strains

The effect of 5-KF on the growth behavior of *E. coli* DSM 498 and *B. subtilis* DSM 10 was investigated by plate-reader assisted microplate cultivation. Cultures were grown in covered 48-well plates (Greiner CELLSTAR^®^ multi-well plates for suspension culture) using a Tecan Infinite M200 plate reader (Tecan Group AG, Männedorf, Switzerland). Strains were cultivated in a 700-μL scale using Wilms-MOPS-medium or Spore minimal medium supplemented with varying concentrations of 5-KF (1 mM, 10 mM, and 20 mM). A 5-KF solution was produced for this purpose using resting cells of *G. oxydans fdh* (section “Production and Purification of 5-Keto-D-Fructose”). The required volume of 5-KF stock solution (158.8 mM) was added to 542.5 μL of the corresponding medium and filled up to 700 μL with H_2_O_*demin*_.

Plate reader cultivation was performed at 30^°^C in the case of *B. subtilis* DSM 10 and 37^°^C in the case of *E. coli* DSM 498. In cycles of 20 min, the plates were shaken linearly for 5 min with a shaking amplitude of 3 mm before detecting the optical density at 600 nm. To compensate for the backscatter effect of the OD_600_ measurement in undiluted cultures during plate reader cultivation, OD calibrations were performed for both strains. For this purpose, the OD_600_ values of several culture dilutions were measured in the plate reader setup and at appropriate dilutions (OD_600_ < 0.3) in a benchtop photometer. The OD_600_ values of the photometer were then plotted against the values of the plate reader, and the calibration equations were determined *via* quadratic regression through the zero point.

Due to a strong Maillard reaction observed in the cultures of *E. coli* DSM 498, the plate reader setup could not assess OD_600_ values. To circumvent this problem, cultures were centrifuged (8,000 × *g*, 20^°^C, 1 min) at the end of the incubation and washed twice in buffer W (100 mM Tris-HCl, 150 mM NaCl, 1 mM EDTA, pH 8). Since the washing volume was equal to the culture volume, the optical density could subsequently be measured at 600 nm.

### Production and Purification of 5-Keto-D-Fructose

To produce 5-KF from D-fructose, a cryopreserved stock of *G. oxydans fdh* was streaked on YMF-plates (section “Shake Flask Cultivation of Microbial Strains”) containing 1.5% [w/v] Agar-Agar, Kobe I, and the antibiotics cefoxitin and kanamycin at final concentrations of 50 μg mL^–1^. To evaluate the impact of potential cellular contamination during 5-KF production on subsequent toxicological studies, a reference fructose solution was prepared using *G. oxydans* 621H Δ*hsdR*, which cannot oxidize D-fructose in the form of dormant cells. Thus, apart from potential contaminations with cellular components, a D-fructose solution emerges unchanged from whole-cell catalysis with resting cells of *G. oxydans* 621H Δ*hsdR*. After 48 h of incubation at 30^°^C, single colonies of each strain were used to inoculate 50 mL of YMF-precultures supplemented with the required antibiotics (see section “Shake Flask Cultivation of Microbial Strains”). Precultures were maintained in shake flasks at 30^°^C and 200 rpm for 48 h for the subsequent inoculation of the main cultures. Therefore, 250 mL of YMF medium were inoculated with 12.5 mL of preculture and incubated for 24 h at 30^°^C and 200 rpm. The cultivation was carried out in 2-L shake flasks to ensure sufficient oxygen supply. After cultures were harvested by centrifugation (8,000 × *g*, 10^°^C, 15 min), cell pellets were resuspended in 10 mL of 50 mM D-fructose solution and again centrifuged, applying the same conditions. This washing procedure was repeated two more times before the cells were finally resuspended in 5 ml of 50 mM D-fructose solution. Bioconversion of D-fructose to 5-KF was carried out at a 250-mL scale in 2-L shake flasks, using a 200 mM D-fructose solution adjusted to pH 6 by adding 5 mM of MES-buffer (pH 6). The washed cells of *G. oxydans* 621H Δ*hsdR* and *G. oxydans fdh* were added to separate flasks and incubated for 18 h at 30^°^C and 200 rpm. After cell removal by centrifugation (10,000 × g, 20^°^C, 20 min), the supernatants were treated with activated charcoal (Cabot Norit GAC 1240 Plus; Cabot Corporation, US, Boston). Therefore, 26 mg of activated charcoal were added per mL supernatant and incubated under stirring for 1 h at 22^°^C. Subsequently, the suspension was subjected to a final centrifugation step (10,000 × *g*, 20^°^C, 10 min). The generated supernatants were first filtered through CHROMAFIL^®^ RC-45/25 syringe filters (0.45 μm pore size) and sterilized by subsequent filtration using sterile PVDF syringe filters (0.22 μm pore size). Filtrates were stored at 8^°^C in sterile falcon tubes.

### Chromatographic Analysis of 5-Keto-D-Fructose and D-Fructose Solutions Produced by Resting Cells of *Gluconobacter oxydans* 621H Strains

For qualitative and quantitative analysis of the prepared 5-KF and D-fructose solution, a Knauer Smartline HPLC-system (Knauer GmbH, Berlin, Germany) was used. The system was composed of a degasser (Knauer Smartline manager 5000), a pump (Knauer Smartline pump 1000), an autosampler (Knauer Smartline autosampler 3800), a column oven (Knauer Column-Thermostat Jetstream 2 Plus), an RI detector (AZURA RID 2.1L), and an ultraviolet (UV) detector (Knauer Smartline UV detector 2600), which measured the absorbance at a wavelength of 210 nm. Sample separation was achieved using the Eurokat H column (300 × 8 mm; Knauer GmbH) and a precolumn (30 × 8 mm; Knauer GmbH) heated to 65^°^C. The mobile phase, 5 mM H_2_SO_4_, was applied at a flow rate of 0.6 mL min^–1^. The injection volume was set to 20 μL. Data evaluation and control of the HPLC system were accomplished using ClarityChrom^®^ 8.2.3 (Knauer GmbH). Peak assignment and quantification were performed by applying external standards of D-fructose and 5-KF in concentrations of 0.5, 1, and 2 mM.

### Ultraviolet-Vis Spectroscopy of 5-Keto-D-Fructose and D-Fructose Solutions Produced by Resting Cells of *Gluconobacter oxydans* 621H Strains

The produced and purified 5-KF solution was analyzed by UV-Vis spectroscopy using a JASCO V-650 spectrophotometer (JASCO Deutschland GmbH, Pfungstadt, Germany). Therefore, 1 ml of a diluted 5-KF solution (final concentration: 15.8 mM) was measured using a bandwith of 1.0 nm between a wavelength of 200 and 700 nm. The measurement was performed in an Eppendorf Uvette^®^ (Eppendorf, Hamburg, Germany), which served as a blank in the empty state.

### Endotoxin Quantification

The endotoxin levels in the prepared 5-KF and D-fructose solutions were quantified using the ToxinSensor Chromogenic LAL Endotoxin Assay Kit (Genscript Biotech, New Jersey, United States). The quantification was performed according to the manufacturer’s instructions.

### Toxicological Studies

#### MTT-Assay

To assess the cell viability of HT-29 cells in the presence or absence of 5-KF, the MTT assay was performed. Therefore, HT-29 cells (10,000/well) were plated in a 96-well plate and supplemented with 200 μL DMEM high glucose medium containing 10% fetal bovine serum (FBS) and antibiotic–antimycotic. Using six biological replicates, cells were supplemented with H_2_O_*pure*_ (negative control), different concentrations of bortezomib (BTZ; positive control), 5-KF, or D-fructose. The latter two compounds were custom-made by whole-cell catalysis using dormant cells of *G. oxydans* 621H Δ*hsdR* and *G. oxydans fdh*. Following incubation at 37^°^C for 18 h in a humidified CO_2_ (5%)/air (95%) atmosphere (referred to as 5% CO_2_ atmosphere), the medium was removed and HT-29 cells were washed with PBS. 3-(4,5-dimethylthiazol-2-yl)-2,5-Diphenyltetrazolium bromide (MTT) was solved in sterile DMEM high glucose medium and added to each well at a concentration of 0.5 mg mL^–1^. After 1 h of incubation at 37^°^C in a humidified 5% CO_2_ atmosphere, MTT was removed and formazan crystals were resolved. Therefore, 100 μL of solubilizing solution (99.5% isopropanol, 0.4% 1 M HCl, and 0.1% Triton X-100) were added and incubated at 37^°^C until formazan crystals disappeared. The absorbance was measured at 570 nm using a Tecan Infinite 200 M Plex plate reader (Tecan Group AG).

### RealTime-Glo™ MT Cell Viability Assay

Prior to the RealTime-Glo™ MT cell viability assay (Promega Corporation, Madison, US), HT-29 cells (1,500/well) were plated in a 96-well plate supplemented with 200 μL DMEM high glucose medium containing 10% fetal bovine serum (FBS) and antibiotic–antimycotic. The plate was incubated for 12 h at 37^°^C in a humidified 5% CO_2_ atmosphere to promote cell adherence. Test compounds (bortezomib, 5-KF, and D-fructose) were diluted in a medium at a concentration 2-fold higher than the target concentration desired in the subsequent assay. The RealTime-Glo reagent (Promega Corp.) was prepared as a 2x stock in the test compound diluent. Following incubation, the medium was removed and the cells were treated with 100 μL of medium containing the respective test compound and 100 μL test compound diluent containing the 2x RealTime-Glo™ reagent. Afterward, cells were incubated at 37^°^C in a humidified 5% CO_2_ atmosphere. In a discontinuous mode, the luminescence of each well was measured at 37^°^C using a Tecan Infinite 200 M Plex plate reader (Tecan Group AG). Per test compound, six biological replicates were performed.

### Photometric Determination of 5-Keto-D-Fructose Induced Maillard Reaction

During the growth studies performed, intensive brown coloring was observed in *E. coli* DSM 498 cultures supplemented with 5-KF, indicating the formation of Maillard products. To investigate the impact of specific media components on the formation of Maillard products, photometric assays were performed in 96-well microtiter plates. Per well, 150 μL H_2_O_*demin*_ was mixed with 7.5 μL Tris-HCl (1 M, pH 7), 30 μL of different dilutions (undiluted, 1:2, 1:4, 1:8, 1:16, and 1:32) of 0.4 M potassium phosphate, 24 μL of different dilutions (undiluted, 1:2, 1:4, 1:8, 1:16, and 1:32) of 0.5 M ammonium chloride, and 15 μL of 99 mM 5-KF solution. The plate was then incubated at 37^°^C in a Tecan Infinite M200 plate reader (Tecan Group AG), which measured the absorbance at 360 nm every 15 min for 20 h. The same experiment was repeated to investigate the impact of L-lysine on the formation of Maillard products in the presence of 5-KF. For this purpose, the experiment was repeated; however, the ammonium chloride solution was substituted with a 0.5 M L-lysine monohydrate solution.

To investigate the formation of fluorescent Maillard products, assays containing 150 μL H_2_O_*demin*_, 7.5 μL Tris-HCl (1 M, pH 7), 30 μL 0.4 M potassium phosphate, 24 μL 0.5 M ammonium chloride, and 15 μL 99 mM 5-KF were transferred to a 96 well plate and subjected to fluorescence spectroscopy. In control assays, 5-KF was substituted with H_2_O_*demin*_. Fluorescence was detected using an excitation wavelength of 365 nm and an emission wavelength of 445 nm. Incubation at 37^°^C and simultaneous measurement of the relative fluorescence units (RLUs) was achieved by an Infinite M Plex plate reader (Tecan AG Group). The experiment was conducted using biological triplicates.

### Data Evaluation, Visualization, and Statistical Analysis

HPLC chromatograms, UV-spectra, and bacterial growth curves were visualized using the scientific plotting package Veusz 3.3.1 (Max-Planck-Institut für extraterrestische Physik, Garching, Germany). Bar charts and *X*–*Y* plots generated to present results of toxicological assays and studies on Maillard reaction were prepared using GraphPad Prism 9.3.1 (GraphPad Software, San Diego, US). Statistical analyses were performed using GraphPad Prism 9.3.1 (GraphPad Software). Assuming equal standard deviations, data obtained during growth experiments with *B. subtilis* DSM 10 and *E. coli* DSM 498 were tested by ordinary one-way analysis of variance (ANOVA). Applying Dunnett’s multiple comparisons test, the means of individual data sets were compared to the mean of the respective water controls. Assuming equal standard deviations, data obtained during toxicological studies on HT-29 cells were tested by ordinary one-way ANOVA. Applying Tukey’s multiple comparisons test, the means of the individual conditions were compared with the means of every other condition.

## Results

### Production of 5-Keto-D-Fructose and D-Fructose by Resting Cells of *Gluconobacter oxydans* 621H *fdh* and *Gluconobacter oxydans* 621H

**FIGURE 1:**
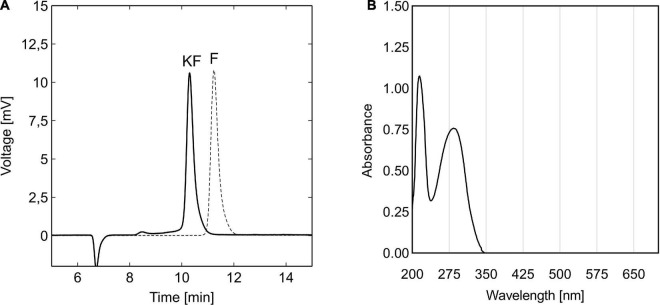
HPLC chromatograms of 5-KF and D-fructose solutions **(A)** and UV-Vis spectrum of 5-KF **(B)** produced by resting cells of *G. Gluconobacter oxydans* 621H strains. After bioconversion of 200 mM D-fructose solution with *G. oxydans fdh* and *G. oxydans* 621H and subsequent downstream processing, final process solutions were analyzed by HPLC and UV-Vis spectroscopy. Chromatographic separation of 5-KF solution (solid line) and D-fructose solution (dotted line) was achieved using the Eurokat H column (300 × 8 mm; Knauer GmbH) heated to 65^°^C. The mobile phase, 5 mM H_2_SO_4_, was applied at a flow rate of 0.6 ml min^–1^. Shown are the signals of the refractive index detector. UV-Vis spectroscopy was performed solely for 5-KF using a JASCO V-650 spectrophotometer (JASCO Deutschland GmbH).

To evaluate the impact of the potential sugar substitute 5-KF on the viability of prokaryotic and eukaryotic cells, the diketone was produced from D-fructose using resting cells of *G. oxydans fdh*. A reference solution of D-fructose, incubated with the wild type strain *G. oxydans* 621H, served as a control to assess the effect of potential microbial contaminations during the production process on toxicological assays. After completion of the bioconversion and subsequent downstream processing, both solutions were analyzed by HPLC, revealing that D-fructose was efficiently oxidized to 5-KF by resting the cells of *G. oxydans fdh* (Figure 1A). No residual substrate was detectable in the final 5-KF solution, confirming the functionality of the production process. However, a tiny preceding peak was detected with a retention time of 8.3 min, most likely caused by a dimerized spirane structure of two 5-KF monomers (β-pyranose and β-furanose). Nonetheless, based on the chromatographic analyses, the purity of the 5-KF solution was found to be > 98%. The use of activated charcoal as an adsorbent thus proved to be extremely effective in removing potential by-products of the bioconversion. During the bioconversion with the wild type strain *G. oxydans* 621H, D-fructose remained untouched and a clean D-fructose peak was observed in the corresponding HPLC chromatogram (Figure 1A).

During UV-Vis spectroscopy, two clear absorption maxima were detected at 286 and 215 nm for the 5-KF solution (Figure 1B). Above a wavelength of 345 nm, no absorption was detected.

Using the ToxinSensor™ Chromogenic LAL Endotoxin Assay Kit (Genscript Biotech), it was confirmed that no microbial endotoxins were present in the prepared 5-KF and D-fructose solutions (data not shown).

### Effect of 5-Keto-D-Fructose on the Viability of Prokaryotic Microorganisms

Growth experiments using *B. subtilis* DSM 10 and *E. coli* DSM 498 were designed to elucidate the effect of 5-KF on the viability of prokaryotic cells. Plate-reader-assisted cultivation in microtiter plates enabled high-throughput assessment of the optical densities at an exceptionally high measurement frequency.

**FIGURE 2:**
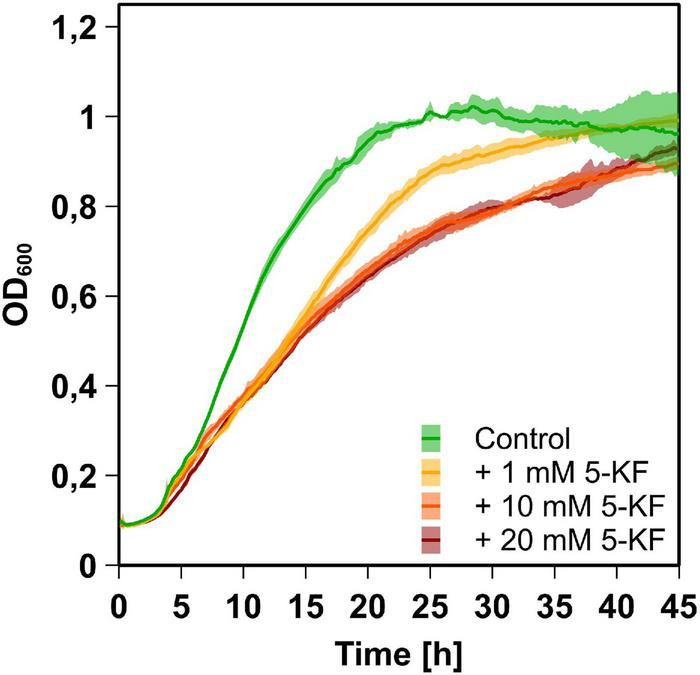
Growth behavior of *B. subtilis* DSM 10 cultures supplemented with and without 5-KF. *B. subtilis* DSM 10 was grown in Spore minimal medium (Demain, 1958) at 30^°^C without 5-KF (green), or with 1 mM (yellow), 10 mM (orange), or 20 mM (red) 5-KF. OD_600_ was measured every 5 min by a Tecan Infinite M200 plate reader (Tecan Group AG). The dark, central line of the growth curves reflects the mean of each biological triplicate, while the lighter area above and below displays the standard deviation. After 28.5 h of incubation, when the control culture reached a maximum OD_600_, all cultures treated with 5-KF displayed significantly lower optical densities (*p* < 0.0001). Dunnett’s multiple comparisons test was performed using GraphPad 9.3.1 (GraphPad Software, San Diego, US) to determine statistical significance.

In Spore minimal medium (Demain, 1958), *B. subtilis* DSM 10 exhibited typical bacterial growth, characterized by an initial lag phase, an exponential growth phase, and a stationary growth plateau (Figure 2). During the initial growth phase (2–7 h), doubling times of all cultures were comparable and ranged from 175 ± 2 to 230 ± 14 min. Subsequently, only the negative control lacking 5-KF maintained its high growth rate, reaching a maximum OD_600_ of 1.31 ± 0.03 after 28.5 h of incubation (Figure 2). Following the initial growth phase, cultures containing 5-KF showed elevated doubling times of 368 ± 13 (1 mM 5-KF), 453 ± 26 (10 mM 5-KF), and 444 ± 7 min (20 mM 5-KF).

**FIGURE 3:**
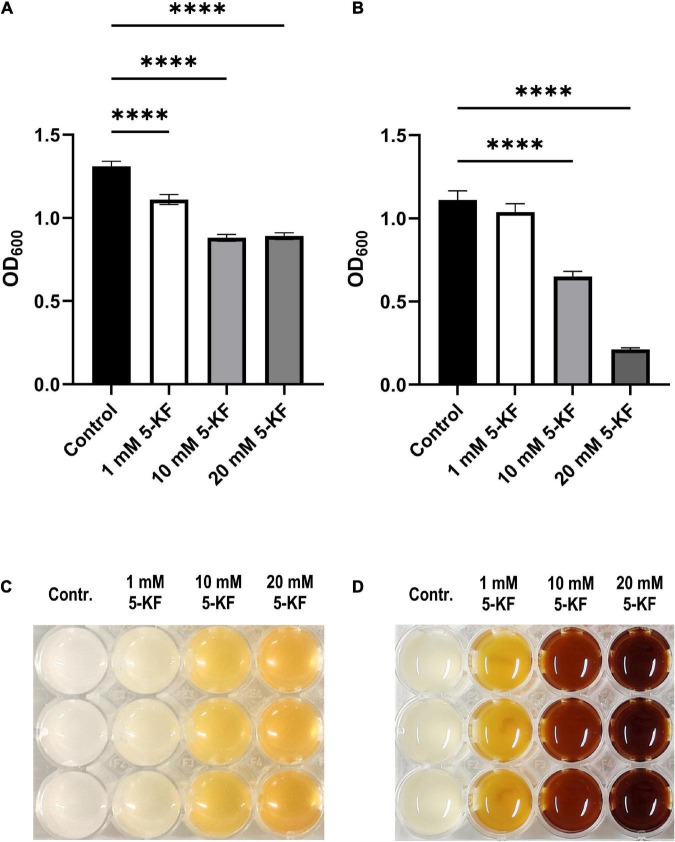
OD_600_ values and browning of *B. subtilis* DSM 10 and *E. coli* DSM 498 cultures treated with and without 5-KF. *B. subtilis* DSM 10 was cultured in Spore minimal medium (Demain, 1958) at 30^°^C without and with different concentrations of 5-KF **(A,C)**. OD_600_-values of *B. subtilis* DSM 10 cultures **(A)** were subjected to statistical analysis after 28.5 h of incubation when the control culture reached its maximum optical density. *B. subtilis* DSM 10 cultures were photographed at the end of the experiment after 45 h of incubation **(C)**. *E. coli* DSM 498 was grown in modified Wilms-MOPS-medium (Wilms et al., 2001) at 37^°^C without and with different concentrations of 5-KF **(B,D)**. Due to the intense browning, the biomass of *E. coli* DSM 498 was not accurately detected by the Tecan Infinite M200 plate reader (Tecan Group AG). After 45 h of incubation, *E. coli* DSM 498 cultures were photographed **(D)**, harvested, washed twice in buffer W, and subjected to final OD_600_ measurement **(B)**. Dunnett’s multiple comparisons test was performed using GraphPad 9.3.1 (GraphPad Software, San Diego, US) to determine statistical significance; ^****^p < 0.0001. The experiment was performed as a biological triplicate.

Accordingly, after 28.5 h of incubation, significantly (*p* < 0.0001) lower OD_600_-values of 1.11 ± 0.03 (1 mM 5-KF), 0.88 ± 0.02 (10 mM 5-KF), and 0.89 ± 0.02 (20 mM 5-KF) were detected in wells supplemented with 5-KF (Figure 3). Although cultures supplemented with 5-KF exhibited a reduced growth rate during the exponential growth phase, they reached similar optical densities as the negative control over the course of the 45-h experiment (Figure 2).

During growth studies with *E. coli* DSM 498, a strong browning of cultures supplemented with 5-KF was observed (Figure 3D). This browning reaction was concentration-dependent and also occurred in a non-inoculated medium (Supplementary Figure 1), indicating reactivity between 5-KF and media components. This observation occurred exclusively with Wilms-MOPS-medium as non-inoculated Spore minimal medium displayed only a faint coloring, which did not affect the assessment of the bacterial biomass (Supplementary Figure 1). Due to the intense brown coloring in cultures of *E. coli* DSM 498, the optical density was not correctly detected by the plate reader platform used. To assess the viability and biomass yields of the cultures, cells were harvested by centrifugation after 45 h of incubation and washed twice in buffer W after discarding the browned supernatants. While the control culture reached an OD_600_ of 1.11 ± 0.06, cultures supplemented with 5-KF displayed decreased maximum optical densities (Figure 3B). This effect was concentration-dependent and significant (*p* < 0.0001) for cultures supplemented with 10 mM and 20 mM 5-KF.

Unlike in the case of *B. subtilis* DSM 10, increasing the 5-KF concentration from 10 to 20 mM had a detrimental effect on the final optical density and thus on the viability of *E. coli* DSM 498. The lowest OD_600_ was observed in the presence of 20 mM 5-KF with 0.21 ± 0.01.

### Effect of 5-Keto-D-Fructose on the Viability of Eukaryotic Cells

The effect of 5-KF on eukaryotic cell viability was investigated using the MTT assay and the RealTime-Glo™ MT Cell Viability assay. The first assay is based on the production of purple-colored formazan crystals from MTT by mitochondrial dehydrogenases of vital cells (Mosmann, 1983). In the second method, a membrane-permeable precursor substrate is reduced by vital cells. In reduced form, the said substrate can be converted by a NanoLuc^®^ luciferase, generating a detectable luminescence signal (Riss et al., 2004). While the MTT assay only allows endpoint determination of cell viability, the RealTime-Glo MT Cell Viability assay can be measured in real-time due to the biocompatibility of the assay components.

#### Assessment of HT-29 Cell Viability in the Presence of 5-Keto-D-Fructose Using the MTT Assay

**FIGURE 4:**
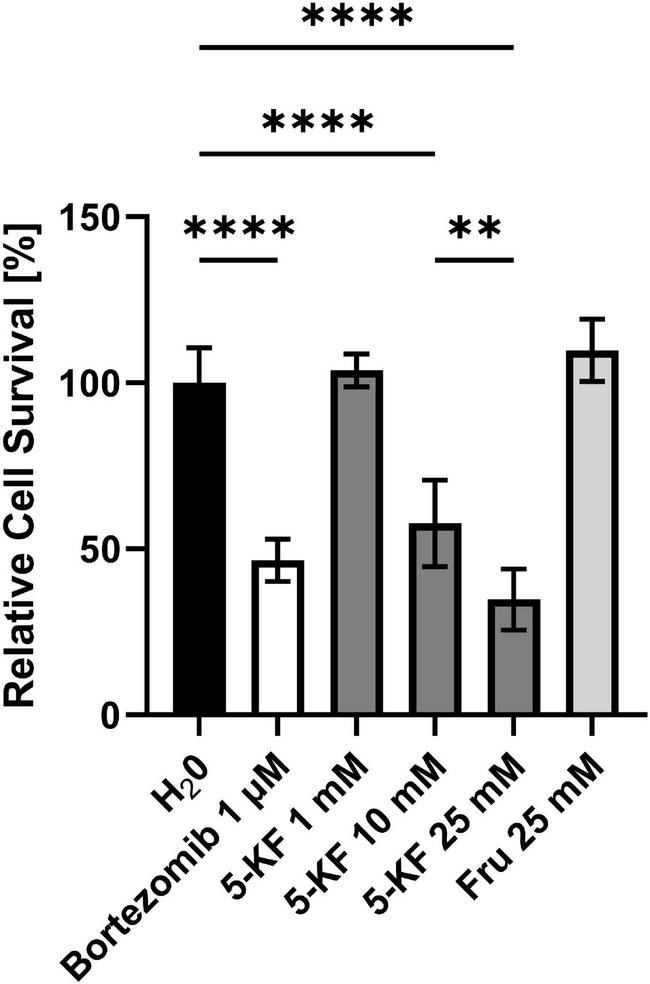
Relative cell survival of HT-29 cells subjected to the MTT assay. The relative cell survival displayed is based on the absorbance caused by formazan crystals produced by HT-29 cells during the MTT assay. HT-29 cells (10,000 cells per well) were incubated in DMEM high glucose medium supplemented with 10% fetal bovine serum (FBS) and antibiotic–antimycotic at 37^°^C in a humidified 5% CO_2_ atmosphere. Cells were supplemented either with H_2_O_*pure*_ or various concentrations of bortezomib, 5-KF, or D-fructose. Once sufficient confluence was reached, the medium was removed from the 96-well plate and MTT was added to each well. The absorbance in each well, caused by redissolved formazan crystals, was measured at 570 nm using a Tecan Infinite 200 M Plex plate reader (Tecan Gr oup AG). Tukey’s multiple comparison test was performed using GraphPad 9.3.1 (GraphPad Software, San Diego, US) to determine statistical significance; ^****^p < 0.0001; ^**^p = 0.023. The experiment was performed using six biological replicates for each test compound.

The absorbance values detected following the MTT assay were used to determine the relative cell survival of the different approaches compared to control assays treated with H_2_O_*pure*_ (Figure 4). Thereby, it was shown that the apoptosis inducer bortezomib led to a significant reduction (*p* < 0.0001) of cell viability at a concentration of 0.01 μM. The relative cell survival in corresponding wells averaged 33.6 ± 5.6%. Increasing the bortezomib concentration to 100 μM further reduced cell viability, as reflected by a relative absorption intensity of 26.6 ± 6.5% (not shown). While wells supplemented with 5-KF at a concentration of 1 mM showed cell viability comparable to the water control (98.8 ± 8.0%), higher 5-KF concentrations reduced the relative cell survival of HT-29 cells significantly (*p* < 0.0001). Cells treated with 10 mM 5-KF displayed a mean relative absorbance of 43.9 ± 5.6%. The inhibitory effect of 5-KF was further enhanced at 25 mM, as the relative cell survival was decreased to 23.6 ± 3.7% in respective assays. Thus, based on these measurements, cells were more restricted in viability in the presence of 25 mM 5-KF than in the presence of 100 μM bortezomib. In contrast, the D-fructose solution incubated with resting cells of *G. oxydans* 621H Δ*hsdR* showed no drastic effect on the viability of HT-29 cells.

#### Assessment of HT-29 Cell Viability in the Presence of 5-Keto-D-Fructose Using the RealTime-Glo™ MT Cell Viability Assay

**FIGURE 5:**
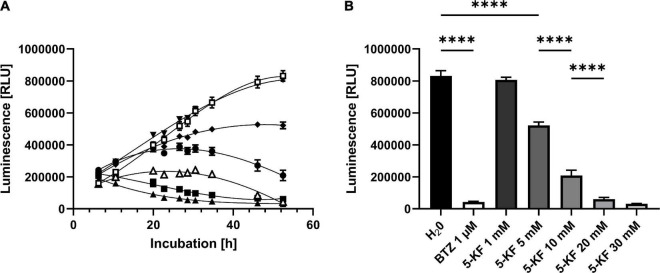
Relative luminescence units (RLUs) produced by HT-29 cells during **(A)** and at the end **(B)** of Promega’s RealTime-Glo™ MT Cell Viability assay. HT-29 cells (1,500 cells per well) were incubated in DMEM High glucose medium supplemented with 10% fetal bovine serum (FBS) and antibiotic–antimycotic at 37^°^C in a humidified 5% CO_2_ atmosphere. Cells were supplemented either with H_2_O_*pure*_ (□), bortezomib (△), or varying concentrations of 5-KF (▼1 mM, ◆ 5 mM, ⬤ 10 mM, ■ 20 mM, ▲ 30 mM). Reagents of Promega’s RealTime-Glo™ MT cell viability assay were added according to the manufacturer’s instructions. At 10 individual time points, the relative luminescence units (RLUs) were assessed using a Promega Glomax^®^ Discover System. Tukey’s multiple comparison test was performed using GraphPad 9.3.1 (GraphPad Software, San Diego, US) to determine statistical significance; ^****^p < 0.0001. The experiment was performed using six biological replicates for each test compound.

To validate the inhibitory effect of 5-KF observed during the MTT assay, the RealTime-Glo™ MT Cell Viability assay (Promega Corp.) was performed. The assay enables real-time determination of luminescence units generated by a NanoLuc**^®^** luciferase upon conversion of a NanoLuc**^®^** Substrate. Said NanoLuc**^®^** Substrate must be generated beforehand by vital cells through reduction of the MT Cell Viability Substrate. Control wells supplemented with H_2_O_*pure*_ displayed a linear increase in luminescence within the first 35 h of incubation (Figure 5A). Subsequently, the increase in luminescence flattened and reached a maximum of 831,533 ± 32,590 relative luminescence units (RLUs) after 52.5 h of incubation (Figure 5B). The addition of 1 μM bortezomib resulted in a significant decrease in luminescence in corresponding wells (*p* < 0.0001). After an initial moderate increase in luminescence in wells treated with bortezomib, the maximal RLU (242,633 ± 5,553 RLUs) of respective wells was measured after 30.5 h of incubation. Toward the end of the experiment, the luminescence signal in assays containing bortezomib decreased to 42,741 ± 3,889 RLUs. In the presence of 1 mM 5-KF the HT-29 cells produced 806,216 ± 16,376 RLUs, which is comparable to the water control. However, at concentrations ≥ 5 mM, the sugar derivative induced a significant decrease in generated luminescence in a dose-dependent manner (*p* < 0.0001). The luminescence levels of assays containing 20 and 30 mM 5-KF were even lower than the luminescence level of assays treated with bortezomib (Figure 5A). After 52.5 h of incubation, the lowest RLU count was observed for wells supplemented with 30 mM 5-KF (31,293 ± 1,690 RLUs). Control assays were carried out to exclude the possibility of 5-KF induced chemical reduction of the NanoLuc**^®^** Substrate (Supplementary Figure 2). No relevant luminescence signals were detected in the control assays, despite the presence or absence of 5-KF, indicating that 5-KF did not interfere with the assay components.

### Investigation of 5-Keto-D-Fructose Induced Maillard Reaction

The intense browning of *E. coli* DSM 498 cultures containing Wilms-MOPS medium and 5-KF indicated the formation of Maillard products (section “Effect of 5-KF on the Viability of Prokaryotic Microorganisms”). During prolonged storage of 5-KF under unfavorable conditions (e.g., non-purified crude preparations at room temperature) it was also observed that corresponding solutions changed color from colorless to yellow-brownish. To elucidate this effect in detail, 5-KF was incubated with varying concentrations of phosphate and ammonium chloride, which are known drivers of the Maillard reaction (Amrein et al., 2006; Moldoveanu, 2021). The effect of L-lysine on the browning reaction induced by 5-KF was investigated as well since the amino acid has been shown to promote a profound Maillard reaction in the presence of reducing sugars (Jing and Kitts, 2000; Ajandouz et al., 2001). The browning reaction was monitored at 360 nm as previous experiments showed that when 5-KF was incubated with the amines listed above, a strong absorbance change occurred at this wavelength (Supplementary Figure 3).

**FIGURE 6:**
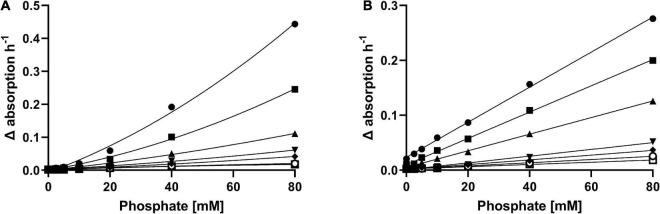
Effect of ammonium chloride **(A)** and L-lysine **(B)** on the Maillard reaction induced by 5-KF and phosphate. Decreasing concentrations (⬤ 80 mM, ■ 40 mM, ▲ 20 mM, ▼ 10 mM, ◆ 5 mM, ○ 2.5 mM, □ 0 mM) of ammonium chloride and L-lysine were incubated with 5-KF and phosphate at 37^°^C. Absorbance at 360 nm was measured every 15 min using a Tecan Infinite M200 plate reader (Tecan Group AG). Shown are the individual absorption changes per hour derived from the performed time-course measurement.

Overall, in the presence of 5-KF all three compounds favored the formation of brown-colored reaction products in a concentration-dependent manner (Figures 6A,B). It was noticeable that in the presence of lower phosphate concentrations, the increase in absorption favored by ammonium chloride was rather small (Figure 6A). However, this trend changed at higher phosphate concentrations (≥40 mM), as the change in absorbance per hour in the presence of ammonium chloride was greater than at equal concentrations of L-lysine. Unlike the experiments performed with ammonium chloride, incubation of L-lysine with 5-KF resulted in a visible increase in absorbance already at low phosphate concentrations. Consecutively, a linear relationship was evident between phosphate concentration, L-lysine concentration, and observed absorbance change per hour.

It is worth noting that in control assays in which 5-KF was substituted with fructose, no change in absorption was observed at 360 nm during the observed reaction period (Supplementary Figure 3). In fact, the UV spectra of a reaction containing D-fructose, phosphate, and ammonium chloride were almost identical at the beginning of incubation and after 16 h of incubation at 37^°^C.

**FIGURE 7:**
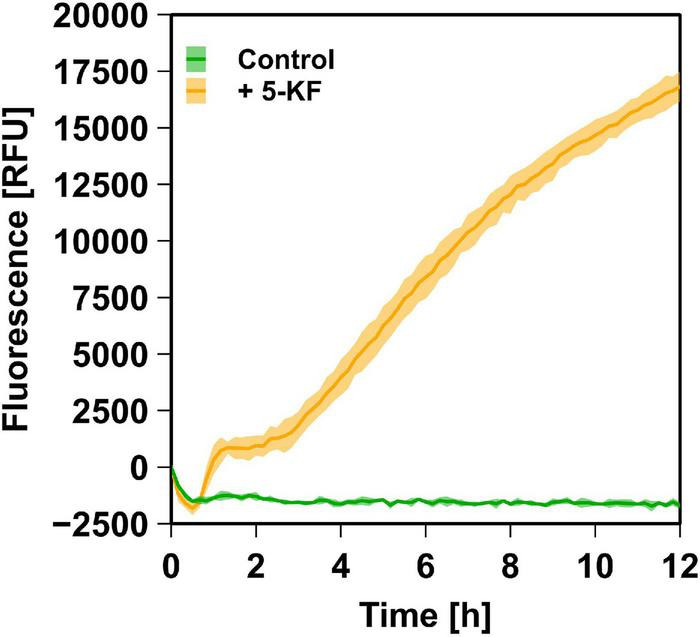
Relative fluorescence units (RFUs) detected in assays containing 5-KF, ammonium chloride, and phosphate. Using 80 mM ammonium chloride, 80 mM phosphate, and 6.45 mM 5-KF fluorescence was measured using an excitation wavelength of 365 nm and an emission wavelength of 445 nm (orange line). In control assays, 5-KF was substituted with H_2_O_*demin*_ (green line). Incubation of the biological triplicates at 37^°^C and simultaneous measurement was achieved using an Infinite M Plex plate reader (Tecan AG Group). The dark, central line of the data shown reflects the mean of each biological triplicate, while the lighter area above and below indicates the standard deviation.

Since the Maillard reaction is accompanied by the formation of several fluorescent compounds (Adhikari and Tappel, 1973; Baker and Bradford, 1994; Leclère and Birlouez-Aragon, 2001), it was examined whether 5-KF promotes the formation of specific fluorophores. These studies were performed using the ammonium chloride/phosphate system due to the stronger brown coloration and absorption changes caused by 5-KF in previous experiments. The assays revealed that in the presence of ammonium chloride and phosphate, 5-KF triggered the formation of fluorescent compounds (Figure 7). In contrast, no increase in fluorescence was observed in control experiments in which 5-KF was replaced by water.

## Discussion

To assess the effect of the potential sugar substitute 5-keto-D-fructose on the viability of prokaryotic and eukaryotic cells, the diketone was produced, purified, and utilized for dedicated growth experiments and toxicological studies.

Oxidation of D-fructose by resting cells of *G. oxydans fdh* and subsequent downstream-processing granted access to a 5-KF solution with very high purity (> 98%). The dominant 5-KF peak in the corresponding HPLC chromatogram showed some minor front tailing and merged into a tiny preceding peak with a retention time of 8.5 min (Figure 1A). While in aqueous solution, 5-KF is present as a *gem*-diol hydrate, and the crystal structure of 5-KF has been reported to be a dimer of linked β-pyranose and β-furanose (Blanchard et al., 1982). This unusual spirane structure was also detected when 5-KF was solved in anhydrous Me_2_SO-*d*_6_, indicating that under anhydrous conditions, 5-KF turns into a dimer (Blanchard et al., 1982). Due to the size-exclusion character of the chromatography column used and the anhydrous character of the mobile phase acetonitrile, it can be suspected that the tiny leading peak was caused by dimerized 5-KF. Incubation of D-fructose with resting cells of the wild-type strain *G. oxydans* 621H was intended to generate a D-fructose solution that would serve as a control during toxicological studies. Since the *fdh*_*SCL*_ gene cluster, encoding the fructose-dehydrogenase complex, is not present in the genome of *G. oxydans* 621H, resting cells of the acetic acid bacterium should not be able to metabolize or oxidize D-fructose. Accordingly, D-fructose was not converted during bioconversion as indicated by the chromatographic HPLC analysis (Figure 1A). These results confirm the observation of Hoffmann et al. (2020), who did not detect measurable Fdh activity in resting cells of *G. oxydans* 621H.

During UV-Vis spectroscopy, two distinct peaks were observed for 5-KF with absorption maxima at 286 and 215 nm (Figure 1B). These peaks come very close to the UV-absorption of isolated keto groups, characterized by an *n* →π* and a π→π* transition. In the case of non-terminal keto groups, as present within 5-KF, the *n* →π* transition exhibits a λ_*max*_ of 273, while the π→π* transition features a λ_*max*_ of 187 nm (Bienz et al., 2016). Thus, according to the conducted UV-Vis spectroscopy, the investigated 5-KF preparation contained the expected keto groups. This finding, however, contradicts the observation, that in water 5-KF predominantly (> 95%) exists in a β-pyranose form, with the 5-keto group being hydrated to form a *gem*-diol (Blanchard et al., 1982). It could be hypothesized that the absorption in the longer wavelength region around 300 nm was caused by residues of the open-chain 5-KF form.

Growth experiments involving the gram-positive and gram-negative bacteria *B. subtilis* DSM 10 and *E. coli* DSM 498 revealed that 5-KF significantly inhibited the growth of both prokaryotes at concentrations ≥ 5 mM (*p* < 0.0001). While this effect was rather moderate but still significant in the case of *B. subtilis* DSM 10 (Figures 2, 3A), a drastic reduction in cell viability was observed for *E. coli* DSM 498 cultures supplemented with 5-KF (Figure 3B). The profound inhibition of *E. coli* DSM 498 correlated with an intense browning of the modified Wilms-MOPS medium (Figure 3D), which also occurred in non-inoculated medium (Supplementary Figure 1). Due to the reducing character of 5-KF, it was assumed that the observed browning was caused by the Maillard reaction. During this process, which is named after the French chemist Louis Maillard (1912), reducing sugars condense with compounds harboring free amino groups to give condensation products (N-substituted sugar-amines), which rearrange to form the so called Amadori rearrangement product (ARP) (Martins et al., 2000). Depending on the pH, this ARP further reacts into reductones, fission products, and Schiff’s bases of hydroxymethylfurfural (HMF) or furfural. In the case of the glucose/glycine Maillard reaction a pH < 5 favors the formation of formic acid and various melanoidins, while at pH > 7 the ARP is mainly converted to flavor compounds (Tressl et al., 1995). Studies have shown that reducing sugars react not only with amino acids but also with ammonia to form a variety of Maillard products, including brown-colored polymers (Hodge, 1953; Van Soest and Mason, 1991). Therefore, it can be assumed that the intense browning observed during the cultivation of *E. coli* DSM 498 was caused by the high ammonium concentration of the Wilms-MOPS-medium and the increased incubation temperature of 37^°^C. The basic prerequisite for ammonium-based Maillard reaction is the deprotonation of ammonium, which takes place spontaneously at elevated temperatures and at neutral, preferably alkaline pH (Huang and Shang, 2006). The ammonia formed under these conditions is even more reactive than amino acids when reacting with reducing sugars (Moldoveanu, 2021). Maillard condensation products of glucose–ammonia systems were shown to occur even at mildly elevated temperatures, leading to the formation of 2,6-deoxyfructosazine and brown-colored polymers (Moldoveanu, 2021). It can be assumed that the much lower concentration of ammonium in the Spore minimal medium and the lower temperature during cultivation of *B. subtilis* DSM 10 were insufficient to promote the formation of ammonia and subsequent Maillard products. Accordingly, the inhibition of *B. subtilis* DSM 10 was much weaker compared to *E. coli* DSM 498.

The inhibitory effect of 5-KF was not limited to prokaryotic cells as the diketone also reduced the viability of the eukaryotic cell line HT-29 in a concentration-dependent manner. At 5-KF concentrations ≥ 5 mM, this effect was significant (*p* < 0.0001) and evident in both the MTT assay (Figure 4) and the RealTime-Glo MT cell viability assay (Figure 5). D-Fructose solution incubated with wild-type *G. oxydans* 621H showed no effect on cell viability, suggesting that no toxic cellular components entered the sugar solutions during microbial bioconversion. Thus, the reduction in cell viability was selectively mediated by 5-KF and not by potentially toxic cell compartments.

Incubation with phosphate and ammonium chloride or L-lysine demonstrated that 5-KF triggered the formation of brown-colored reaction products, as confirmed by a substantial increase in absorbance at 360 nm (Figure 6). The accelerating effect of phosphate on Maillard browning is well-known but the exact mechanism for phosphate involvement is still not clear (Rizzi, 2004). The anion might act as a catalyst for the formation of reactive dicarbonyl derivatives as key intermediates of the Maillard reaction and facilitates the nucleophilic attack of amines on the keto groups of 5-KF. The observed increase in absorbance change at elevated concentrations of ammonium chloride or L-lysine is also consistent with observations in the literature (Amrein et al., 2006; Moldoveanu, 2021). The assumption that 5-KF favors the formation of Maillard products was supported by fluorescence measurements. Fluorescent products have been proposed as specific indicators for the Maillard reaction (Adhikari and Tappel, 1973; Baker and Bradford, 1994; Leclère and Birlouez-Aragon, 2001) and were also detected in assays containing 5-KF, phosphate, and ammonium chloride (Figure 7). Several studies reported on fluorescent compounds detected during the Maillard reaction, which had a maximum of excitation at wavelengths between 340 and 370 nm and showed maximum emission at wavelengths between 420 and 450 nm (Morales and Van Boekel, 1998; Leclère and Birlouez-Aragon, 2001; Rufian-Henares et al., 2002).

Based on the experiments conducted, it cannot be definitively elucidated whether the cytotoxic effect of 5-KF originates from the diketone itself or from the Maillard products formed in the presence of 5-KF. Since Maillard products have been shown to possess mutagenic and cytotoxic properties (Brands et al., 2000; Jing and Kitts, 2000), it is likely that the observed inhibition of *B. subtilis* DSM 10 and *E. coli* DSM 498 was induced by toxic Maillard products. Nonetheless, the rapid onset of inhibition observed during growth experiments with *B. subtilis* DSM 10 (Figure 2) and toxicological studies with HT-29 cells (Figure 5) indicate that 5-KF may also directly impact cellular processes in a detrimental way.

Due to the inhibitory effects of 5-KF toward prokaryotic and eukaryotic cells, we advise against using 5-KF in the food sector as a sugar substitute. Further studies are recommended to substantiate the effect shown here. Nevertheless, the high reactivity of the compound could be of great use in other branches of research, such as organic chemistry.

## Conclusion

Due to the increasing number of sugar-associated diseases and the growing consumer awareness of the adverse effects of sugar, there is an urgent need for healthy sugar alternatives. A promising candidate with this regard is 5-keto-D-fructose, a natural diketone, which is produced in some acetic acid bacteria by oxidation of D-fructose, a process catalyzed primarily by the membrane-bound fructose dehydrogenase complex. In this work, the effect of 5-KF on prokaryotic and eukaryotic cells was investigated. It was shown that 5-KF significantly reduced the viability of *B. subtilis* DSM 10, *E. coli* DSM 498, and the epithelial cell line HT-29 at concentrations ≥ 5 mM. This effect might be related to the strong Maillard reaction favored by 5-KF in the presence of amines, which became apparent during this study. Due to the cytotoxic effects of the diketone, we hereby oppose the use of 5-KF in the food sector.

## Data Availability Statement

The raw data supporting the conclusions of this article will be made available by the authors, without undue reservation.

## Author Contributions

MH: conceptualization, validation, visualization, investigation, supervision, and writing—original draft. NG: investigation and methodology. SK, AK, JS, and TL: investigation. KK: conceptualization, writing—review and editing. UD: conceptualization, project administration, resources, funding acquisition, supervision, and writing—review and editing. All authors contributed to the article and approved the submitted version.

## Conflict of Interest

The authors declare that the research was conducted in the absence of any commercial or financial relationships that could be construed as a potential conflict of interest.

## Publisher’s Note

All claims expressed in this article are solely those of the authors and do not necessarily represent those of their affiliated organizations, or those of the publisher, the editors and the reviewers. Any product that may be evaluated in this article, or claim that may be made by its manufacturer, is not guaranteed or endorsed by the publisher.
