# Supplementary material for: 5-Keto-D-Fructose, a Natural Diketone and Potential Sugar Substitute, Significantly Reduces the Viability of Prokaryotic and Eukaryotic Cells
Source: Front Microbiol. 2022 Jun 21;13:935062. doi: 10.3389/fmicb.2022.935062 (PMC9253636; doi:10.3389/fmicb.2022.935062)
Supplement: Supplementary file 1 [file Data_Sheet_1.docx]

**Supplementary material**

**Front. Microbiol. Fig. 1S**


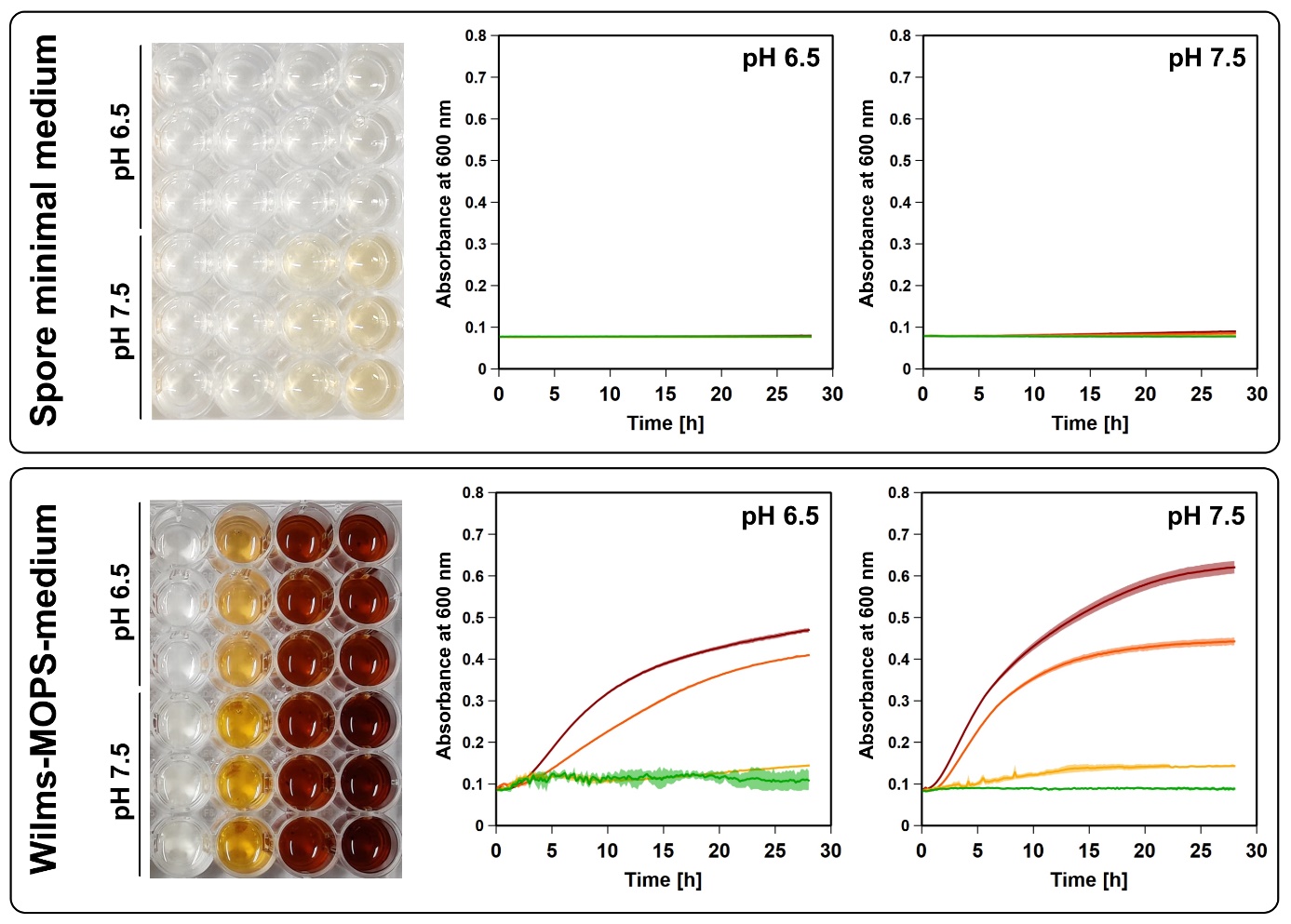


Figure 1S: Macroscopic images and absorbance of non-inoculated Spore minimal medium (upper box) and Wilms-MOPS-medium (lower box) supplemented with 5-KF adjusted to pH 6.5 and pH 7.5. The macroscopic images were taken after 45 hours of incubation at 30 °C (Spore minimal medium) and 37 °C (Wilms-MOPS-medium). The three upper rows of the 48 well plates were adjusted to pH 6.5 the lower three rows to pH 7.5. Absorbance (600 nm) of each well was measured without 5-KF (1^st^ column, green), or with 1 mM (2^nd^ column, yellow), 10 mM (3^rd^ column, orange), or 20 mM (4^th^ column, red) 5-KF. Incubation and photometric measurements were achieved using a Tecan Infinite M200 plate reader (Tecan Group AG). The dark, central line of the OD_600_ graphs reflects the mean of each biological triplicate, while the lighter area above and below displays the standard deviation.

**Front. Microbiol. Fig. 2S**


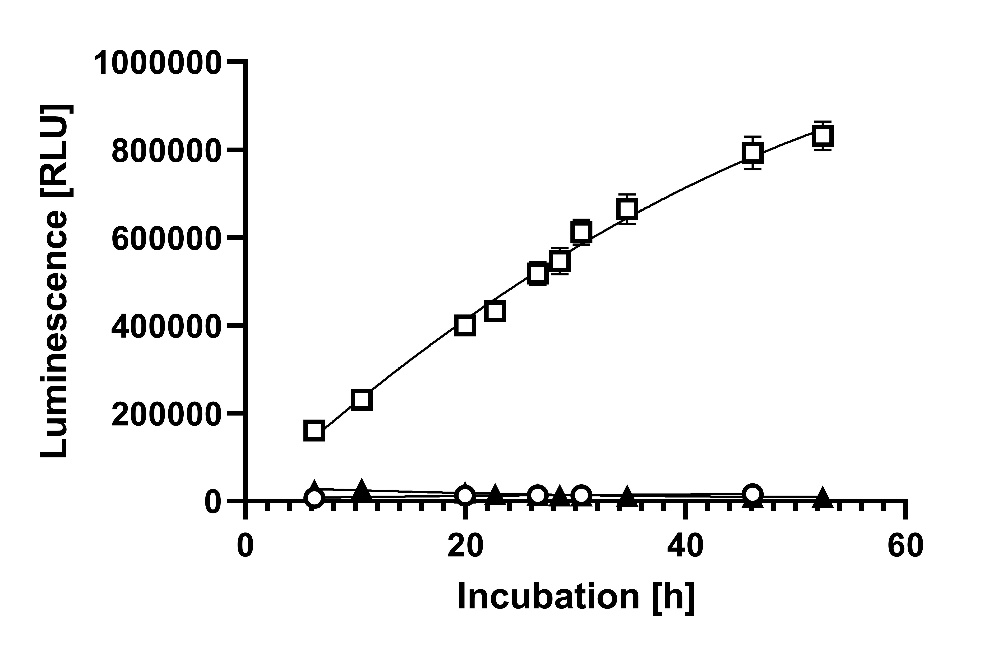


Figure 2S: Relative luminescence units (RLUs) detected in control assays during the RealTime-Glo™ MT Cell Viability assay. HT-29 cells (1,500 cells per well) were incubated in DMEM High glucose medium supplemented with 10 % fetal bovine serum (FBS) and Antibiotic-Antimycotic at 37 °C in a humidified 5 % CO_2_ atmosphere. Reagents of the RealTime-Glo™ MT Cell Viability Assay (Promega Corp.) were added according to the manufacturer's instructions. At ten individual time points, the relative luminescence units (RLUs) were assessed using a Promega Glomax^®^ Discover System (Promega Corp.). Displayed are the RLUs detected within a control assay supplemented with H_2_O_r_ (□), a control lacking HT-29 cells (○), and a control lacking HT-29 cells which was supplemented with 30 mM 5-KF (▲). To improve the presentation of the data, every second data point of the assay lacking HT-29 cells (○) was manually removed. The experiment was performed using six biological replicates for each test compound.

**Front. Microbiol. Fig. 3S**


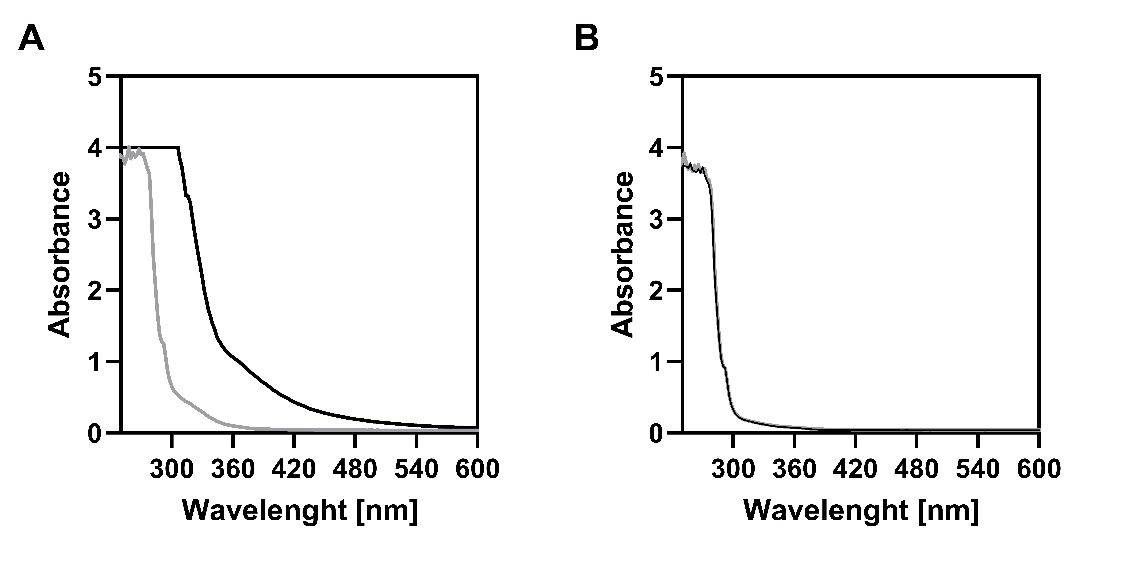


Figure 3S: UV-Vis spectra of 5-KF (A) and d-fructose solutions (B) incubated with ammonium chloride and phosphate. Both assays were carried out at a 220 µL scale in a 96 well plate and contained 7.5 µL Tris-HCl (1 M; pH 7), 30 µL potassium phosphate (400 mM), 25 µL ammonium phosphate (500 mM) and either 7.5 µL 5-KF (186 mM) or 7.5 µL d-fructose solution (186 mM). The reactions were finally made up to 220 µL with H_2_O_demin_. At the beginning of the experiment (grey lines) and after 16 hours (black lines) of incubation at 37 °C the absorbance of each well was measured by a Tecan Infinite M200 plate reader (Tecan Group AG). The maximum absorbance that could be detected by the instrument was 4.
